# Reactivation of *M. tuberculosis* Infection in Trans-Membrane Tumour Necrosis Factor Mice

**DOI:** 10.1371/journal.pone.0025121

**Published:** 2011-11-21

**Authors:** Ivy Dambuza, Roanne Keeton, Nasiema Allie, Nai-Jen Hsu, Philippa Randall, Boipelo Sebesho, Lizette Fick, Valerie J. F. Quesniaux, Muazzam Jacobs

**Affiliations:** 1 Division of Immunology, Institute of Infectious Disease and Molecular Medicine, University of Cape Town, Cape Town, South Africa; 2 CNRS UMR6218, Orleans, France; 3 Molecular Immunology and Embryology, University of Orleans, Orleans, France; 4 National Health Laboratory Service, Sandringham, South Africa; French National Centre for Scientific Research, France

## Abstract

Of those individuals who are infected with *M. tuberculosis*, 90% do not develop active disease and represents a large reservoir of *M. tuberculosis* with the potential for reactivation of infection. Sustained TNF expression is required for containment of persistent infection and TNF neutralization leads to tuberculosis reactivation. In this study, we investigated the contribution of soluble TNF (solTNF) and transmembrane TNF (Tm-TNF) in immune responses generated against reactivating tuberculosis. In a chemotherapy induced tuberculosis reactivation model, mice were challenged by aerosol inhalation infection with low dose *M. tuberculosis* for three weeks to establish infection followed chemotherapeutic treatment for six weeks, after which therapy was terminated and tuberculosis reactivation investigated. We demonstrate that complete absence of TNF results in host susceptibility to *M. tuberculosis* reactivation in the presence of established mycobacteria-specific adaptive immunity with mice displaying unrestricted bacilli growth and diffused granuloma structures compared to WT control mice. Interestingly, bacterial re-emergence is contained in Tm-TNF mice during the initial phases of tuberculosis reactivation, indicating that Tm-TNF sustains immune pressure as in WT mice. However, Tm-TNF mice show susceptibility to long term *M. tuberculosis* reactivation associated with uncontrolled influx of leukocytes in the lungs and reduced IL-12p70, IFNγ and IL-10, enlarged granuloma structures, and failure to contain mycobacterial replication relative to WT mice. In conclusion, we demonstrate that both solTNF and Tm-TNF are required for maintaining immune pressure to contain reactivating *M. tuberculosis* bacilli even after mycobacteria-specific immunity has been established.

## Introduction

Although a third of the global population has been exposed to tuberculosis the majority harbours a latent form of infection [1]. This global reservoir potential poses significant challenges to therapeutic intervention, made more difficult by poor understanding of the immune mechanisms that exert pressure to maintain bacilli in a state of latency. Real threats are associated with disease reactivation, particularly in disease burden countries in which immune-compromised individuals such as those with HIV/AIDS form a significant part of the population. In low burden, first world countries, reactivation of latent bacilli form the primary cause of active disease as opposed to new infections in developing countries.

Host immune factors that allow for mycobacteria to remain in a persistent state of latency have not been clearly defined although considerable insight has been gained through the application of *in vitro* models and animal studies that simulate *M. tuberculosis* reactivation [2], [3], [4], [5]. However, identifying factors responsible for maintaining a latent infectious state and those that are compromised to give rise to reactivation have proven to be complex. Loss of function and neutralization studies has been key to understand the effects of Tumour Necrosis Factor (TNF) in host protection. We and others have shown that while TNF is critical to control acute infection [6], [7], [8], it is similarly important to prevent bacilli replication during chronic infection [9] or during drug induced latent infection [10]. The reemergence of bacilli in the absence of TNF correlated with a lack of proper granuloma structures and the increase of pro-inflammatory cytokines. The importance of TNF for maintaining latent infection was verified in clinical studies in which anti-TNF therapy administered to patients with chronic inflammatory diseases resulted in spontaneous reactivation of tuberculosis [11], [12], [13], [14]. The mechanisms through which TNF mediates control of latent infection is unclear, however studies have reported that administration of TNF inhibitors interferes with TNF mediated phagosome maturation, apoptosis, T cell activation and autophagy [15]. A study by Bruns et al., 2009 showed that anti-TNF neutralizing antibodies reduced the population of effector memory CD8 T cells resulting in reduced antimicrobial activity against *M. tuberculosis* [16].

TNF is a multifunctional cytokine, initially synthesized as a 26 Kda non-glycosylated type II trans membrane protein (Tm-TNF) which upon cleavage by the metalloprotease TNF-converting enzyme (TACE) forms a soluble 17 KDa protein. Both molecular forms of TNF are biologically functional as homotrimeric proteins that bind and mediate signaling through either TNFRp55 (TNFRSF1A, CD120a,TNFR1) or TNFRp75 (TNFRS1B, CD120b, TNFR2) with Tm-TNF binding strongly TNFRp75 [17]. We and others have previously reported that acute *M. tuberculosis* infection could be controlled by Tm-TNF but that soluble TNF was required to sustain host immune protection [18], [19], [20], [21]. Moreover, we have demonstrated that rapid and lethal reactivation of *M. tuberculosis* was associated with lack of proper bactericidal granuloma formation in latently infected complete TNF^−/−^ mice treated with isoniazid and rifampicin [10].

With the current development of new TNF inhibitor biologics which specifically inhibit solTNF and spare Tm-TNF in the treatment of chronic inflammatory disorders [22], [23], [24], [25], we investigated the role of Tm-TNF in controlling reactivation of therapeutically induced latent infection. We show that Tm-TNF mediates control of reactivating bacilli but that soluble TNF is required to sustain long-term growth inhibition. We found that susceptibility in reactivating Tm-TNF mice is associated with unstructured granuloma formation and a defect of protective cytokine synthesis.

## Materials and Methods

### Mice

C57Bl/6 wild type (WT) control mice, TNF^−/−^ mice [26] and Tm-TNF mice [27] were bred, maintained and housed in individually ventilated cages under specific pathogen free conditions in the animal facility of the University of Cape Town, South Africa. For all the experiments, age matched mice on a C57Bl/6 background were used and genotypes were confirmed by PCR analysis. All the experiments and protocols performed were in accordance with the guidelines of the Research Ethics Committee of the University of Cape Town, South Africa (Approval ID- REF REC: 008/023).

### Bacterial infection and chemotherapy

*M. tuberculosis* H37Rv was grown in Middlesbrook 7H9 broth (Becton, Dickinson and Company, Le Pont de Claix, France) supplemented with 10% Middlebrook OADC enrichment medium (Life Technologies, Gaitherburg, MD), 0.5% glycerol and 0.05% Tween 80 at 37°C until log phase. Prior to usage, mycobacterial aliquots were passed 30× through a 29.5 G needle to minimize bacterial clumping. Pulmonary infection with 100–200 cfu live *M. tuberculosis* H37Rv bacteria was performed using a Glas-Col Inhalation Exposure System, Model A4224. Inoculum size was confirmed 24 h post-infection by determining the bacterial burden in the lungs of infected mice.

**Figure 1:**
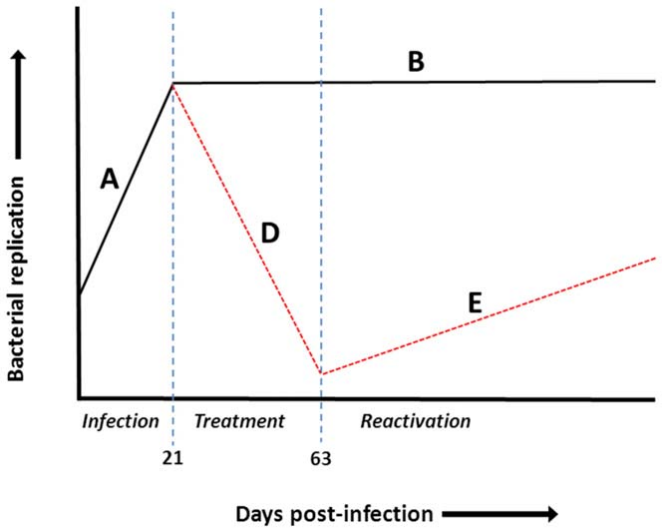
Graphic presentation of drug-based *M. tuberculosis* reactivation mouse model. Line A: Preimmune phase, short bacterial replication period post-infection with low dose *M. tuberculosis*; Line B: Steady state phase: Control of *M. tuberculosis* growth through host immunity and establishment of chronic infection. Line D: Drug treatment phase: Reduction of bacilli replication through INH-RIF chemotherapy. Line E: Reactivation phase: Reactivation of infection upon cessation of antibiotics.

For the *M. tuberculosis* reactivation model (Fig. 1), we used a modified protocol as previously described [10]. Briefly, groups of mice were infected by aerosol inhalation with 100–200 cfu viable *M. tuberculosis* H37Rv bacilli. The infection was allowed to progress for 21 days, termed the pre-immune phase (Fig. 1, line A), where unrestricted bacilli replication occurred before commencement of treatment with 25 mg/Kg INH-RIF (Sigma, St. Louis, USA) in drinking water for 6 weeks. In this drug treatment phase (Fig. 1, line D) bacilli numbers were reduced to at least less than 100 CFUs in the lungs after which treatment was withdrawn, allowing reactivation of bacilli (Fig. 1, line E). Alternatively, in control groups that received no therapy after 21 days, bacilli burdens remained constant (steady state phase Fig. 1, line B).

### Colony enumeration assay

Bacterial burdens in the lungs, livers and spleens of infected mice were determined at specific time points after infection with *M. tuberculosis* H37Rv. Organs were weighed and homogenized in 0.04% Tween 80 saline. Tenfold serial dilution of organ homogenates were plated in duplicates on Middlesbrook 7H10 (Becton, Dickinson and Company) agar plates containing 10% OADC (Life Technologies, Gaitherburg, MD) and incubated at 37°C for 19–21 days. Colonies on plates were enumerated and bacterial burdens determined.

### Pulmonary pathology

Mice were euthanized by carbon dioxide inhalation at specific time points. Organs were weighed and fixed in 10% formalin and paraffin-embedded. Two to 3 µm sections were stained with haematoxylin and eosin (H&E) and a modified Ziehl-Nielson (ZN) method as described [28]. For immunostaining, formalin-fixed paraffin-embedded sections were deparaffinised and rehydrated then stained with rabbit anti-mouse specific inducible nitric oxide synthase (iNOS) [29], rat anti-mouse CD11b (clone M1/70) or rat anti-mouse CD3 (clone 145-2c-11) antibodies. Sections were then washed in PBS and incubated for 30 min at room temperature with biotinylated secondary antibody then subsequently incubated with avidin-biotin complexes (Vector Laboratories, CA, USA) for 30 min, washed and incubated with DAB substrate (Dako Corporation, CA, USA).

### Lung homogenate preparations

Whole lungs were removed from infected mice at specific time points and were homogenized in 1 ml 0.04% Tween 80 saline containing protease inhibitor (Sigma) and the supernatants were collected after low-speed centrifugation, aliquoted and frozen at −80°C.

### Cytokine ELISA

Supernatants from organ homogenates or from cultured cells were harvested and assayed for cytokine concentration using commercially available ELISA reagents for IFNγ, IL-10, and IL-12p70 (R&D Systems, Germany and BD PharMingen, San Diego), according to the manufacturer's instructions.

### Statistical Analysis

The data are expressed as the mean ± SEM. Statistical analysis was performed by ANOVA. For mortality studies, analysis was performed using the log-rank test. For all tests, a p-value of <0.05 was considered significant.

## Results

### Tm-TNF protects mice from severe tuberculosis reactivation

In a study performed by McCune *et al*., 1966, it was observed that immunizing mice with *M. tuberculosis* then re-infecting them preceding treatment with anti-tuberculous drugs resulted in mice reactivating with lower *M. tuberculosis* CFU numbers compared to control groups [30]. This result was interpreted as the influence of the host's acquired immune resistance to mycobacteria. In this study, we investigated the contribution of two molecular forms of TNF, in particular Tm-TNF in host immunity in mice immunized by prior infection with *M. tuberculosis*. The model used entailed aerosol inhalation infection of WT mice, TNF^−/−^ mice and Tm-TNF mice with 100 viable *M. tuberculosis* H37Rv bacilli. The infection was allowed to progress for 21 days before commencement of treatment with 25 mg/Kg INH-RIF in drinking water for 6 weeks to reduce bacilli numbers to at least less than 100 CFUs in the lungs after which treatment was withdrawn and tuberculosis reactivation was monitored. Body weights were recorded throughout the infection period and body weight loss was interpreted as severe disease due to reappearing tuberculosis and correlated with susceptibility to infection. Control groups of WT, Tm-TNF and TNF^−/−^ mice that did not receive chemotherapeutic treatment were confirmed to have phenotypes as previously described [18].

**Figure 2:**
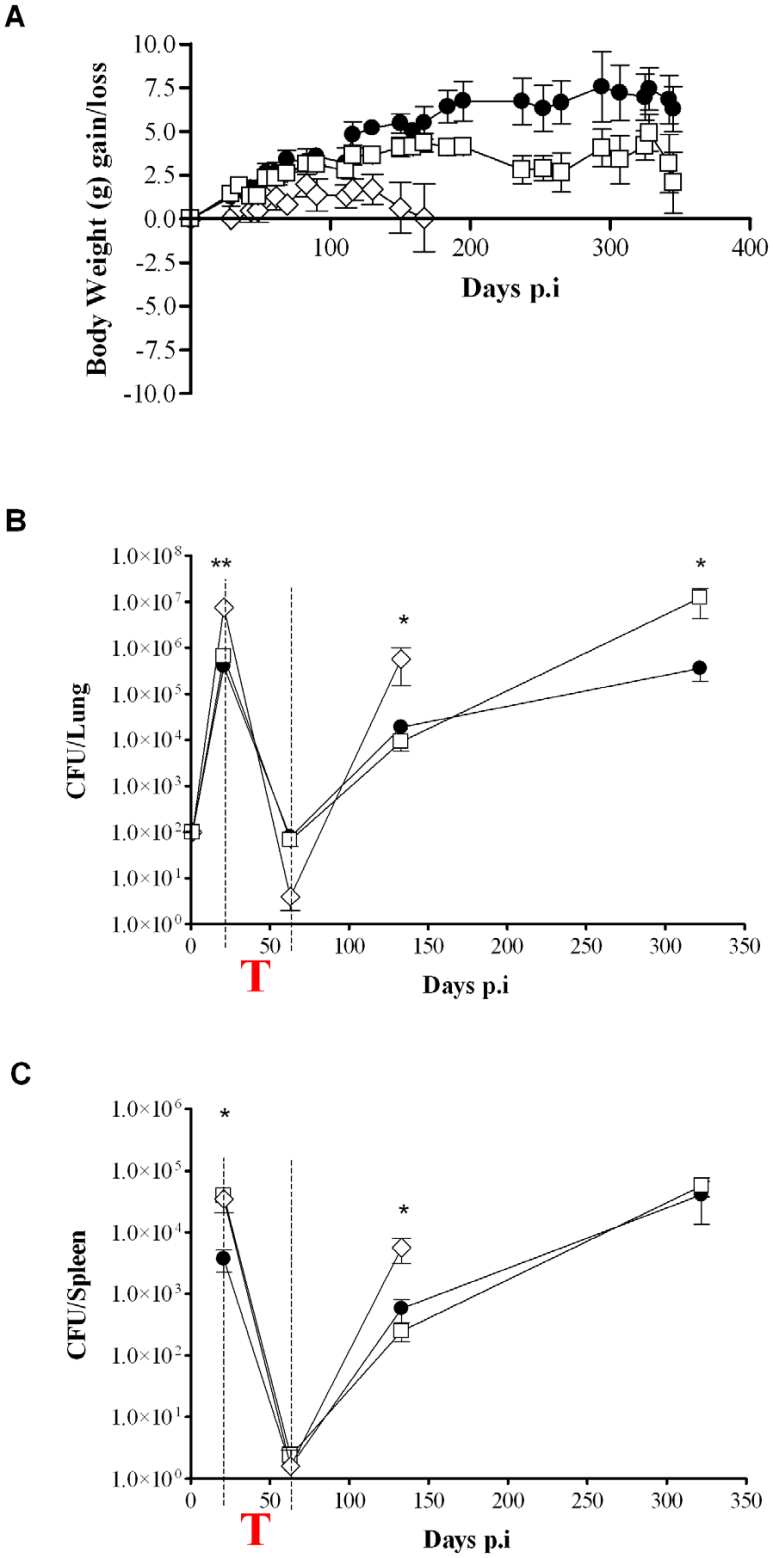
Effect of Tm-TNF on *M. tuberculosis* replication during reactivation. WT mice (black circles), TNF^−/−^ mice (white diamonds) and Tm-TNF mice (white squares) were treated for 6 weeks with 25 mg/Kg INH-RIF in drinking water subsequent to 3 weeks aerosol infection with 100–200 CFU's of *M. tuberculosis* H37Rv. (A) Body weights were recorded throughout the course of the infection period and bacterial burdens in lungs (B) and spleens (C) were enumerated at time points indicated. Data are representative of two experiments and data points are expressed as the mean ± SD of 5 mice/group (for CFUs). The body weight study consisted of between 6–16 mice/group where the data represents weights for remaining mice in each group. The red “T” in the figure corresponds to the drug treatment phase. Significant differences (*p<0.05; **p<0.01) were determined by Student's *t* test for comparisons between two groups and ANOVA for comparisons between three groups.

We found that, of the therapeutically treated animals, WT mice showed a steady increase in body weight over the duration of the infection in contrast to TNF^−/−^ mice which displayed significantly lower body weights between 69 and 165 days post-infection (Fig. 2A) coinciding with the period subsequent to the withdrawal of treatment. TNF^−/−^ mice also appeared sick with ruffled fur and hunched backs and eventually became moribund and had to be terminated on day 165. In contrast, Tm-TNF showed an increase in body weight comparable to WT mice for the first 130 days post-infection (Fig. 2A) but significant weight loss was recorded in Tm-TNF mice between 237 days and 265 days post-infection while the body weights in WT mice remained stable (Fig. 2A). For the remainder of the experimental period, Tm-TNF mice maintained slightly lower body weights relative to WT mice with no significant differences observed (Fig. 2A) but exhibited no physical signs of severe disease apparent in TNF^−/−^ mice. These data indicate that complete absence of TNF renders mice susceptible to severe reactivating tuberculosis which is alleviated by the presence of Tm-TNF.

Next, bacilli burdens in lungs and spleens of infected mice were determined at specific time points to investigate the effect of the different molecular forms of TNF on mycobacteria recrudescence in the presence pre-existing immunity in WT mice, TNF^−/−^ mice and Tm-TNF mice. Bacilli burdens in WT mice were reduced by 3.5 log_10_ in the lung (Fig. 2B) and by 2.5 log_10_ in the spleen (Fig. 2C) after exposure to 6 weeks INH-RIF treatment. Withdrawal of antibiotic treatment resulted in spontaneous *M. tuberculosis* reactivation with bacilli burdens reaching up to 4 log_10_ in the lung and 2.5 log_10_ in the spleen (Fig. 2B & C, respectively). In TNF^−/−^ mice, INH-RIF treatment reduced the high bacilli burden by more than 6 log_10_ in lung (Fig. 2B) and 4 log_10_ in spleen (Fig. 2C) after 6 weeks exposure. The more efficacious reduction in bacterial burden in the lung of TNF^−/−^ relative to WT mice after INH-RIF treatment is in agreement with the concept that reduced immune pressure is intimately associated with improved antibiotic-mediated mycobacterial clearance [31]. Within 133 days post-treatment, mycobacteria reappeared in TNF^−/−^ mice and bacilli burden reached at least 6 log_10_ in lung (Fig. 2B) and 4 log_10_ in spleen (Fig. 2C) of fully deficient TNF^−/−^ mice. Tm-TNF mice responded to INH-RIF treatment in a manner comparable to WT mice and showed a similar slow kinetic in the rate of reactivation comparable to that of WT mice. However, by 322 days post-treatment, bacterial burdens in the lung of Tm-TNF mice increased significantly by 2 log_10_ compared to a 1 log_10_ increase observed in WT mice (Fig. 2B). Bacilli burdens in spleens of Tm-TNF mice were comparable to WT mice at all time points investigated (Fig. 2C). Therefore the data show that Tm-TNF is required to promote early bacterial killing mechanisms even after priming of the host by previous *M. tuberculosis* infection. However, although Tm-TNF confers early protection against recrudescence *M. tuberculosis*, Tm-TNF alone does not sustain long term control of the infection, indicating that solTNF is needed for controlling chronic infection.

### Abnormal inflammatory response in the absence of TNF during tuberculosis reactivation is delayed in Tm-TNF mice

**Figure 3:**
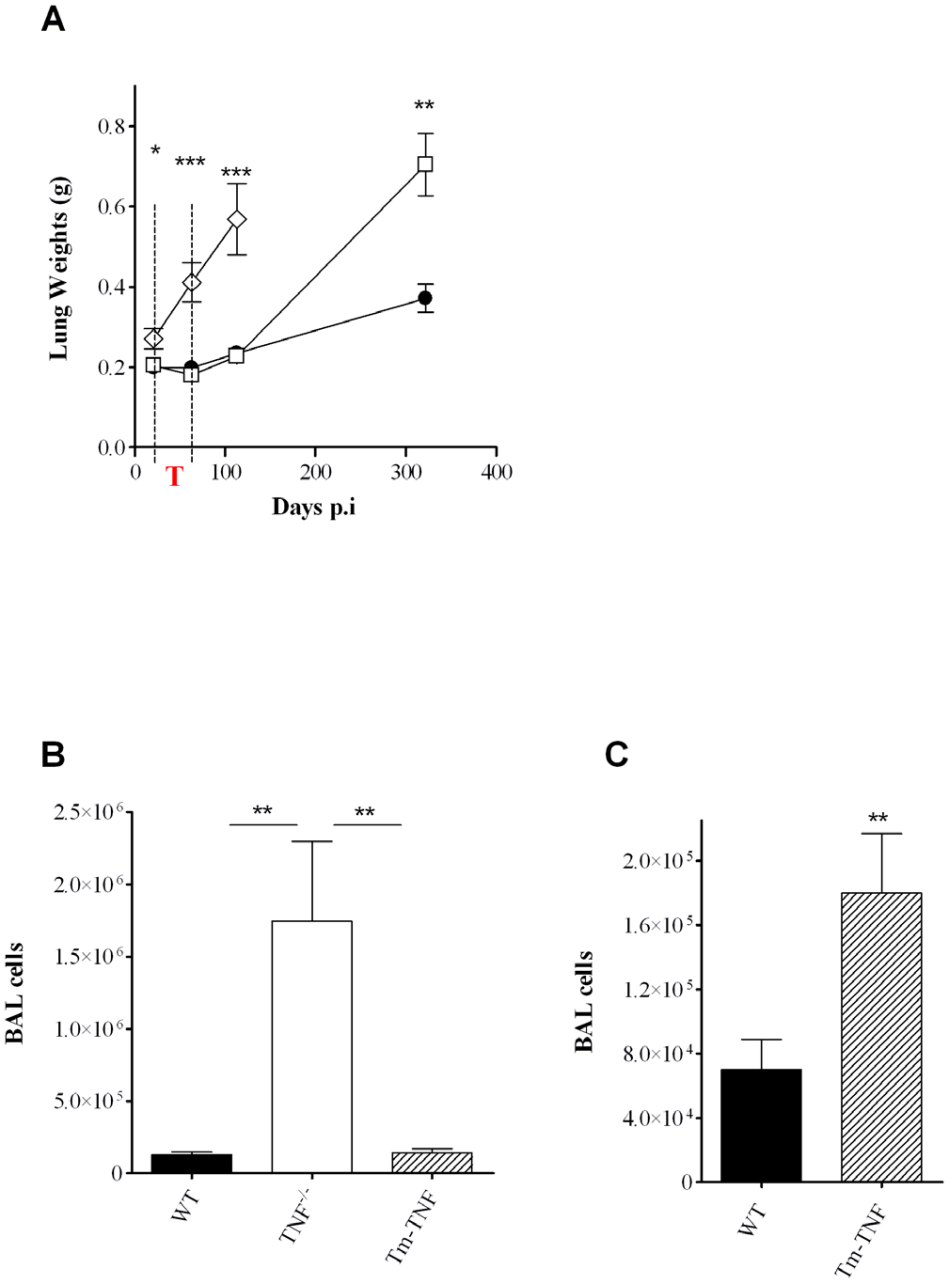
Induction of excessive inflammation in the absence of solTNF during reactivation of *M. tuberculosis*. WT (black circles), TNF^−/−^ (white diamonds) and Tm-TNF (white squares) mice were exposed by aerosol inhalation infection to 100–200 CFUs/mouse of *M. tuberculosis* H37Rv for 3 weeks preceding chemotherapy with 25 mg/Kg INH-RIF for 6 weeks in drinking water. (A) Lung weights were measured at specific time points and BAL derived cell numbers were determined 77 days (B) and 378 days (C) post-infection. The red “T” in the figure corresponds to the drug treatment phase. Data are representative of 1 of 2 experiments performed and are expressed as mean ± SD of 5 mice/group. Significant differences (*p<0.05; **p<0.01; ***p<0.001) were determined by Student's *t* test for comparisons between two groups and ANOVA for comparisons between three groups.

TNF has previously been shown to be at the apex of inflammatory responses [32]. To determine whether Tm-TNF was sufficient in mediating an inflammatory response during tuberculosis reactivation, mouse lung weights were recorded at specific time points during the infection period as a surrogate marker of inflammation. Compared to lung weights determined at day 21 post-infection, WT mice displayed no change in lung weights after exposure to INH-RIF for 6 weeks. However, an increase in lung weights was observed during later stages of disease (322 days post-infection; Fig. 3A) which was consistent with the increase in bacilli burdens at this time point (Fig. 2B). In sharp contrast, by 21 days post-infection, TNF^−/−^ mice already displayed significantly higher lung weights compared to WT mice (Fig. 3A). Paradoxically, 63 days post-infection at the end of the therapy period, the lung weights had increased significantly (*p*<0.001) compared to WT mice (Fig. 3A) but did not correlate with the decreased number of bacilli at this time point (Fig. 2B). This observation differed from a previous report where similar lung weights were measured in TNF^−/−^ mice in a model using a 3 fold lower infection dose and chemotherapy was initiated earlier [10]. We postulate that here, a delay in onset of chemotherapy until 21 days combined with the potential higher antigenic burden of killed bacilli provided conditions for irreversible proinflammatory stimulation and excessive inflammation in the absence of TNF. Susceptibility of TNF^−/−^ mice was confirmed with a further significant increase (*p*<0.001) in lung weights noted at 113 days post-infection, 50 days after cessation of therapy. In contrast, lung inflammation in Tm-TNF mice was similar to WT mice during early infection with similar lung weights at 21, 63 and 133 days post-infection (Fig. 3A). However, control of inflammation was not sustained as a significant increase (*p*<0.01) in lung weights was evident in Tm-TNF mice at day 322 post-infection compared to WT mice (Fig. 2.1B).

To further analyze airways inflammation in the absence of TNF, or in the presence of Tm-TNF, the number of cells present in the bronchoalveolar lavage (BAL) fluid was determined after infection. The cellularity in the BAL fluid was significantly higher in TNF^−/−^ mice compared to WT mice, whereas Tm-TNF mice had a number of cells comparable to WT mice at day 77 post-infection (Fig. 3B) confirming that the control of inflammation during early infection was membrane TNF dependent. Consistent with chronic lung weight data, Tm-TNF mice displayed significantly increased number of cells in BAL fluid relative to WT mice on day 378 post-infection (Fig. 3C). Together, the data indicates that control of early inflammation is mediated primarily by Tm-TNF but solTNF is required for regulation of inflammation during chronic infection. However, the lack of control of inflammation appears to be strongly associated with the onset of tuberculosis reactivation.

### Tm-TNF is inadequate to maintain bactericidal granulomas during reactivating tuberculosis

**Figure 4:**
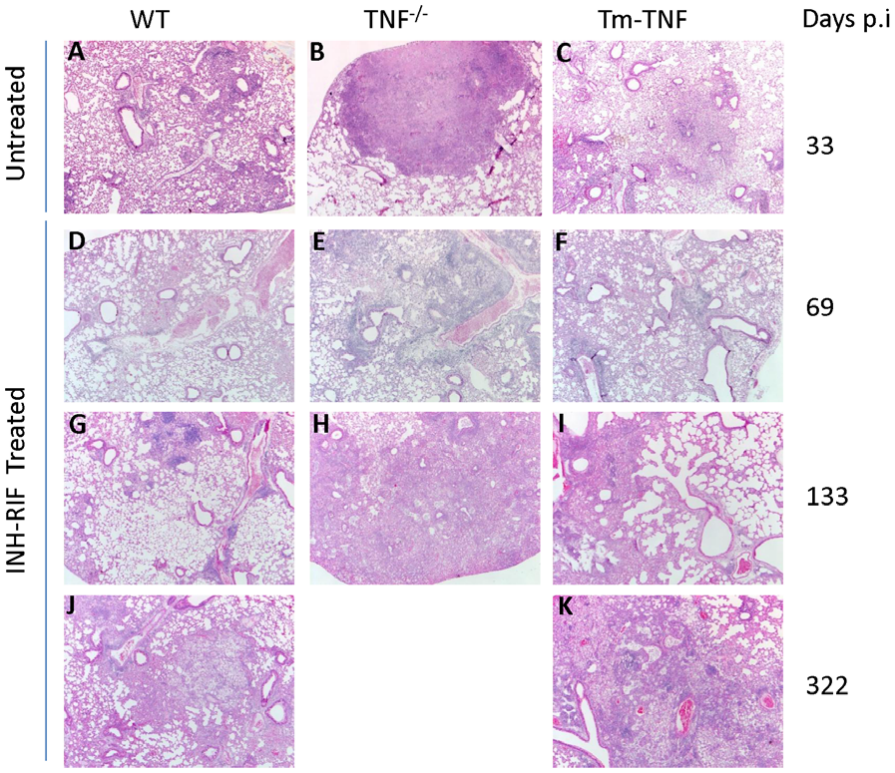
Tm-TNF contributes to protective bactericidal granuloma formation during *M. tuberculosis* reactivation but is insufficient to sustain structural integrity and bactericidal efficacy. WT mice (A,D,G,J), TNF^−/−^ mice (B, E, H) and Tm-TNF mice (C,F,I,K) were infected by aerosol inhalation with 100–200 CFUs/mouse *M. tuberculosis* H37Rv for 3 weeks preceding chemotherapy with 25 mg/Kg INH-RIF for 6 weeks in drinking water. Lungs were removed at the indicated timepoints and tissue sections stained with haematoxylin and eosin to determine the granulomatous response.

We next asked whether granuloma structures were formed in the presence of Tm-TNF during tuberculosis reactivation. Studies performed by Mohan et al., 2001, illustrated that TNF was required for maintenance of granuloma structure during persistent *M. tuberculosis* infection whereby upon treatment with TNF-neutralizing antibody, mice displayed severe histopathology marked with excessive inflammation and loss of structured granulomas [33]. Lung sections were obtained from infected WT mice, TNF^−/−^ mice and Tm-TNF mice at the indicated time points post-infection and pulmonary pathology analyzed. Untreated WT mice (Fig. 4A) and Tm-TNF mice (Fig. 4C) displayed small compact lesions with tight lymphocytic wedges and a high degree of clear airway spaces at day 33 post infection. In sharp contrast, TNF^−/−^ mice (Fig. 4B) showed enlarged unstructured lesions with inflammation occupying larger areas of the lung and presenting with evidence of necrosis. At the end of chemotherapeutic treatment (day 63 p.i.), WT (Fig. 4D) and Tm-TNF (Fig. 4F) mice had pulmonary pathology characterized by well-defined granulomas and clear alveoli whereas TNF^−/−^ mice displayed higher inflammation characterized by peri-vascular and peri-bronchiolar inflammation (Fig. 4E). Development of pathology subsequent to withdrawal of chemotherapy (day 133 p.i.) continued to show well-defined granulomas in WT (Fig. 4G) and Tm-TNF (Fig. 4I) mice with clear alveoli, whereas TNF^−/−^ mice now presented with excessive inflammation with no defined granulomas and limited alveolar space (Fig. 4H). The inability of Tm-TNF mice (Fig. 4K) to control development of further pathology was clearly evident at day 322 post-infection where, in contrast to WT mice (Fig. 4J), Tm-TNF mice displayed enlarged unstructured lesions with excess inflammatory responses and interstitial pneumonia.

These observations demonstrate that during tuberculosis reactivation, Tm-TNF does not sustain long term maintenance of protective granuloma structure in the absence of soluble TNF resulting in malformed lesions that associate with failure to inhibit *M. tuberculosis* growth.

**Figure 5:**
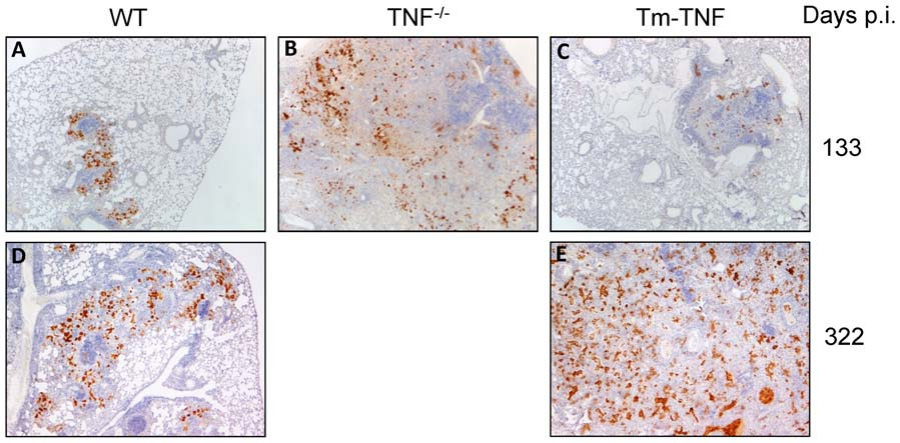
Extra-granulomatous pulmonary expression of INOS is associated with susceptibility in Tm-TNF reactivating mice. WT mice (A,D), TNF^−/−^ mice (B) and Tm-TNF mice (C,E) were infected by aerosol inhalation with 100–200 CFUs/mouse *M. tuberculosis* H37Rv for 3 weeks preceding chemotherapy with 25 mg/Kg INH-RIF for 6 weeks in drinking water. Lungs were removed at 133 and 322 days post infection and tissue sections were stained with polyclonal rabbit anti-mouse antibody (see *Materials and methods*). Brown stain represents iNOS expression by activated macrophages. Micrographs represent 4 animals/group and are shown at ×32 magnification.

**Figure 6:**
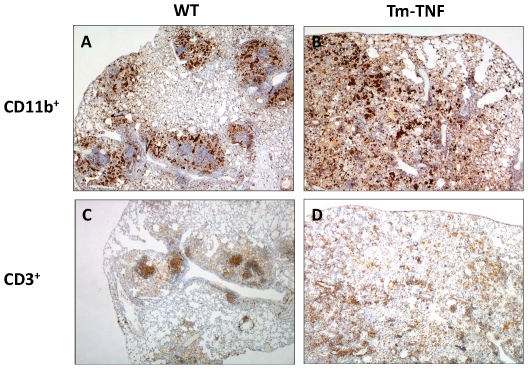
Macrophage (CD11b^+^ cells) and lymphocyte (CD3^+^ cells) recruitment in WT and Tm-TNF mice. WT mice (A,C) and Tm-TNF mice (B,D) were infected by aerosol inhalation with 100–200 CFUs/mouse *M. tuberculosis* H37Rv for 3 weeks preceding chemotherapy with 25 mg/Kg INH-RIF for 6 weeks in drinking water. Lungs were removed 322 days post infection and tissue sections were stained with either anti-CD11b anti-mouse (A,B) antibody or anti-CD3 anti-mouse antibody (C,D) (see Materials and methods). Micrographs represent 4 animals/group and are shown at ×32 magnification.

We therefore determined whether effector macrophage anti-mycobacterial function was intact in Tm-TNF mice undergoing *M. tuberculosis* reactivation. Studies have shown that cell mediated mycobacterial killing function can be achieved through production of toxic reactive intermediates (RNI) via the enzymatic action of macrophage iNOS [34]. Previously, it was demonstrated that inhibition of iNOS in mice chronically infected with *M. tuberculosis* resulted in reactivation of tuberculosis disease with increased organ bacillary burdens and extensive granulomatous response [10], [35]. In view of these findings we determined iNOS expression immunohistochemically in lung tissue sections of reactivating infected WT mice, TNF^−/−^ mice and Tm-TNF mice after completion of chemotherapy. iNOS expression patterns were largely within the confinement of granuloma lesions in WT mice (Fig. 5A) in contrast to TNF^−/−^ mice where the pattern was dispersed and associated with the diffused granuloma lesions (Fig. 5B) analyzed 133 days post-infection. Tm-TNF mice displayed similar iNOS expression to WT mice at day 133 days post-infection (Fig. 5C). However, during reactivating chronic infection at day 322, in contrast to WT mice (Fig. 5D), increased iNOS expression was evident in Tm-TNF mice (Fig. 5E) with a random distribution and evidence of lung tissue destruction. We further characterized the effect of Tm-TNF signaling on macrophage (CD11b^+^ cells) and lymphocyte (CD3^+^ cells) recruitment to investigate whether a correlation existed between macrophage recruitment and iNOS induction, and also to determine Tm-TNF effects on the structural relationship between macrophages and lymphocytes with respect to granuloma formation during reactivation. It is clear that macrophage distribution correlated strongly with iNOS induction where, in WT mice it was localized on the periphery of established granulomas (Fig. 6A) but randomly distributed in Tm-TNF mice (Fig. 6B) at 322 days post-infection. Lymphocytes recruitment was focused and predominantly occupied central areas of granulomas in reactivating WT mice (Fig. 6C) but was unorganized in reactivating Tm-TNF mice (Fig. 6D). Therefore, these observations indicate that, in hosts where tuberculosis reactivation occurs, Tm-TNF on its own cannot sustain the structural integrity of granulomas with respect to the cellular organization of macrophages and lymphocytes, and that iNOS induction by macrophages is insufficient for controlling mycobacterial growth if the granuloma structure is not properly formed.

### Defective protective cytokine induction in reactivating Tm-TNF mice during late stages of the disease

**Figure 7:**
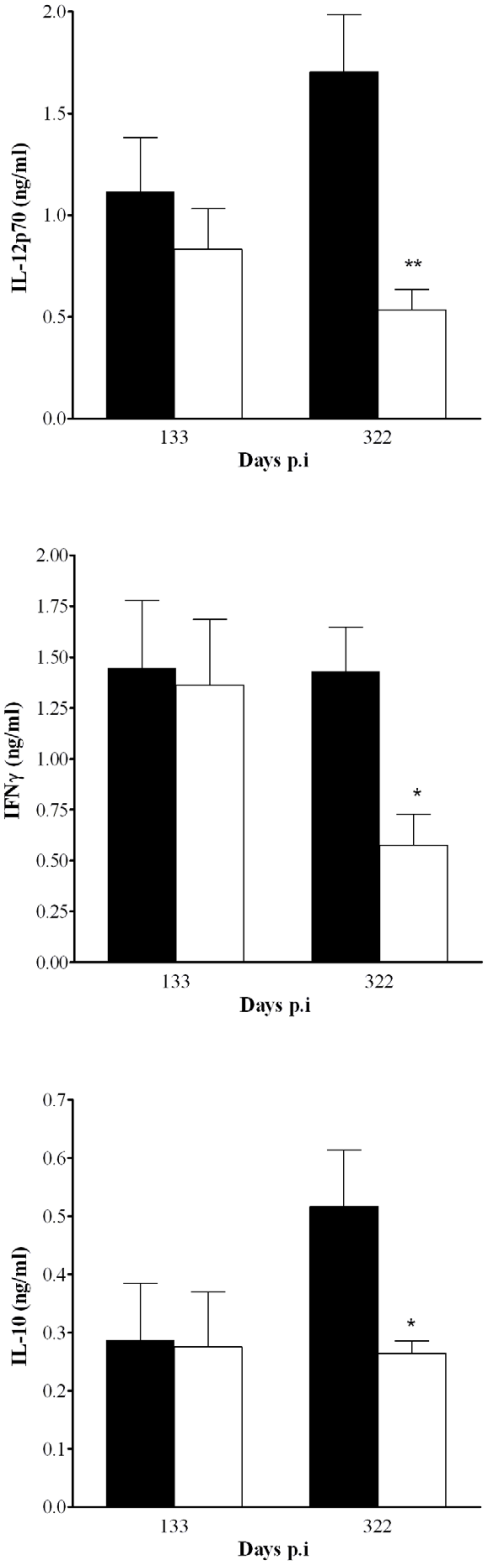
Reduced pulmonary cytokine expression is associated with increased susceptibility in Tm-TNF mice during *M. tuberculosis* reactivation. WT (closed bars) and Tm-TNF mice (open bars) were exposed by aerosol inhalation to 100–200 CFUs/mouse of *M. tuberculosis* H37Rv for 3 weeks preceding chemotherapy with 25 mg/Kg INH-RIF for 6 weeks in drinking water. Lungs were obtained and homogenized at 133 days and 322 days post-infection and the levels of IL-12p70, IFNγ and IL-10 present in cell supernatants determined by ELISA. Data represent 1 of 2 experiments performed and values are expressed as mean ± SD of 5 animals/group. Significant differences (*p<0.05; **p<0.01) were determined by Student's *t* test.

Next we assessed the effect of Tm-TNF mediated immune responses in infected mice subsequent to INH-RIF treatment with particular reference to the quantification of IFNγ and IL-12 levels because of their reported functions in generating and maintaining protective immunity against *M. tuberculosis* infection [36], [37], [38]. Studies by Feng et al., 2005, demonstrated that continuous IL-12 production is necessary for maintenance of pulmonary IFNγ-producing effector CD4^+^ T cells and subsequent bacilli control during chronic *M. tuberculosis* infection suggesting that interruption of IL-12 signal transduction contribute to development of reactivation of tuberculosis [39]. Therefore, we quantified pulmonary IL-12 and IFNγ production during tuberculosis reactivation (Fig. 7). Comparable IL-12p70 and IFNγ concentrations were found in the lung of WT mice and Tm-TNF mice 133 days post-infection. However, there was a significant decrease in IL-12p70 and IFNγ pulmonary concentrations in Tm-TNF compared to WT mice 322 days post-infection associated with susceptibility of Tm-TNF mice to *M. tuberculosis* reactivation at this time point. The concentration of anti-inflammatory IL-10 was comparable 133 days post-infection but increased in WT mice by day 322 post-infection and remained significantly lower in Tm-TNF mice (Fig. 7). These data suggest that Tm-TNF is not sufficient to sustain protective cytokine induction in post-infection *M. tuberculosis* chronic immunity and this phenotype is associated with lack of control of bacilli burden in the lung and lethality of these mice.

## Discussion

Estimates indicate that a third of the global population is latently infected with the tuberculosis pathogen, *M. tuberculosis* [1]. The host mechanisms responsible for maintaining a latent infection remain elusive. Several studies have established that TNF is important in immune responses against mycobacterial infections [6], [28], [40], [41], [42], [43] but it is also a central mediator of pathology in autoimmune diseases [44], [45], [46]. Patients on anti-TNF treatment for chronic inflammatory diseases have an increased incidence of tuberculosis [11], [12], [13], [14] implicating TNF in the preservation from latent tuberculosis. Although highly efficacious, the currently used anti-TNF therapies i.e. etanercept, infliximab, and adalimumab block both Tm-TNF and solTNF [47], [48], [49], [50]. Therefore, further research on TNF biology is required for designing improved therapeutics that will alleviate inflammatory diseases while maintaining protection against mycobacteria.

We and others investigated the contribution made by the two molecular forms of TNF, solTNF and Tm-TNF, in the induction of protective immunity or immunopathology during *M. tuberculosis* or *M. bovis* BCG infection [18], [19], [20], [51]. A conclusion derived from these studies revealed that Tm-TNF expressing mice have an intermediary phenotype i.e. they controlled acute infection where gene deficient TNF mice were susceptible. However Tm-TNF mice succumbed to chronic infection with increased bacillary burdens and excessive granulomatous response which was not observed in WT mice. Here, we report that Tm-TNF and solTNF are both required for the maintenance of immune pressure during reactivating tuberculosis. We found in our drug-induced tuberculosis reactivation model that TNF is an absolute requirement for the containment of the re-emergence of tuberculosis reflected by the rapid propagation of mycobacteria after cessation of antibiotic treatment in TNF^−/−^ mice. This observation confirms findings previously reported by Botha and Ryffel, 2003 [10] and support data showing that treatment of chronically infected mice [33], [52] or latently infected humans treated with TNF neutralizing antibodies results in reappearance of tuberculosis [11], [12], [13], [14], [16], [53], [54]. Furthermore, our results show that Tm-TNF contributes to the initial containment of re-emerging tuberculosis but is not sufficient for long-term mycobacteria containment resembling the outcome of chronic infection. Together, these observations demonstrate that Tm-TNF mediates early protection against reactivating *M. tuberculosis* infection and that solTNF might be required at later stages for sustained protection.

The formation of granulomas in response to mycobacterial challenge leads to killing or alternatively, isolation and confinement of bacilli to local sites of infection. They are dynamic structures that promote cellular interaction to enhance bactericidal efficacy, and the initial establishment and continued maintenance of its structural integrity in murine models is critically dependent on TNF. Nevertheless, the relationship between TNF and granuloma structure as an indicator of protection mediated against infectious mycobacteria remains controversial. More recently, Lin *et al*, 2010 found that neutralization of TNF in cynomolgus macaques resulted in dissemination of disease without compromising the structural integrity of established granulomas [55]. Previously, we proposed that Tm-TNF alone was sufficient to develop distinct granulomas during respiratory *M. tuberculosis* infection, but referred to the diminished bactericidal capacity of such granulomatous structures [18]. Moreover, these findings were supported in clinical studies by Lliopoulos *et al*, 2006 who found typical granulomas in biopsy specimens from patients on anti-TNF therapy who developed tuberculosis [56]. Furthermore, although presence of structurally defined granulomas are widely accepted as the hallmark of protection, Johnson *et al*, 1998 reported adequate protection in ICAM-1 deficient mice despite lacking structured granulomas during *M. tuberculosis* infection [57]. These combined observations therefore suggest that factors independent of TNF may determine granuloma structural integrity and that such structures alone cannot be used as a marker to define protection against *M. tuberculosis*. Here, we show that during tuberculosis reactivation, TNF^−/−^ mice formed larger lesions with inflammation occupying larger areas of the lung with some evidence of necrosis compared to WT mice, an observation that corroborates previous findings by Mohan et al., 2001 and Botha and Ryffel, 2003 where a lack of proper defined granulomas in reactivating TNF^−/−^ mice were described [10], [33]. Furthermore, our results show that Tm-TNF mice were capable of granuloma structure formation during initial tuberculosis reactivation comparable to WT mice but that long term Tm-TNF dependent sustainability was not enough to maintain protective granuloma structures and susceptibility was reflected by formation of larger, more diffuse lesions with excess inflammation and interstitial pneumonia in reactivating mice. These results therefore illustrate a role for both solTNF and Tm-TNF as a requirement for maintaining granuloma structures during tuberculosis reactivation.

We further assessed iNOS induction as a parameter of macrophage activation *in situ* within granulomas to understand the lack of sustained Tm-TNF mediated protection during *M. tuberculosis* reactivation. It is clear that Tm-TNF induced activation of macrophages in a focused manner where iNOS expression was localized to structured granulomas unlike in the complete absence of TNF. The sustainability of this initial focused macrophage response was however compromised during latter stages where iNOS synthesis was notably randomized. The failure to sustain focused bactericidal activity within granulomas was accompanied by a reduction in overall cytokine profile that included IFNγ, IL-12p70 and IL-10. Clinically, similar findings were noted in reactivating tuberculosis patients on anti-TNF therapy who presented with a decreased T cell activation profile and reduction in IFNγ and IL-10 synthesis [58]. Lower IFNγ levels, in particular, critically impacts on macrophage responses by suppressing phagosome maturation and promoting mycobacterial survival. We therefore postulate that, in our studies, the dependence of IL-12p70 synthesis on autologous TNF mediated signaling by antigen presenting cells such as macrophages which are mostly located within granulomas may eventually have been compromised in Tm-TNF deficient mice. The inhibition of T cell activation and lower IFNγ production, as a consequence of diminished IL-12 levels, may have resulted in suppressed *M. tuberculosis* specific phagosome maturation, thereby promoting bacterial survival and replication during long term reactivation. The cytokine profile in reactivating Tm-TNF mice reported here contrasts to that observed in *de novo* infected Tm-TNF mice [18] with respect to pulmonary IFNγ AND IL-10 concentrations during late stage infection despite similar observed susceptible phenotypes. We postulate that cytokine induction under these very diverse conditions may be driven by distinctive differences in immune responses. Under conditions of *de novo M. tuberculosis* infection innate cellular responses drive initial protective immunity whereas under conditions of reactivation protective function may primarily be driven by adaptive memory immunity established during infection prior to pathogen resolution after chemotherapeutic treatment.

In conclusion data presented here, illustrates that TNF mediated immunity against *M. tuberculosis* infection requires both Tm-TNF and solTNF during tuberculosis reactivation. Although Tm-TNF protects mice against acute *M. tuberculosis* infection, long term protection requires solTNF partly to down regulate inflammatory responses in chronic infection and to sustain immune pressure during recrudescence of *M. tuberculosis* infection.
